# Age at natural menopause and associated factors in adult women: Findings from the China Kadoorie Biobank study in Zhejiang rural area

**DOI:** 10.1371/journal.pone.0195658

**Published:** 2018-04-18

**Authors:** Meng Wang, Wei-Wei Gong, Ru-Ying Hu, Hao Wang, Yu Guo, Zheng Bian, Jun Lv, Zheng-Ming Chen, Li-Ming Li, Min Yu

**Affiliations:** 1 Department of NCDs Control and Prevention, Zhejiang Provincial Center for Disease Control and Prevention, Hangzhou, China; 2 Chinese Academy of Medical Sciences, Dong Cheng District, Beijing, China; 3 Department of Epidemiology, School of Public Health, Peking University Health Science Center, Haidian District, Beijing, China; 4 Clinical Trial Service Unit and Epidemiological Studies Unit (CTSU), Nuffield Department of Population Health, University of Oxford, Oxford, United Kingdom; Institute for Nutritional Sciences, CHINA

## Abstract

**Objectives:**

To investigate the factors associated with age at natural menopause in a large population of Chinese adult women.

**Methods:**

This cross-sectional study was part of the baseline survey of China Kadoorie Biobank in Zhejiang Province. A total of 17,076 postmenopausal women were included in the present study. Relevant data of socio-demographic, lifestyle, dietary and reproductive characteristics were collected. Multinomial logistic regression models were used to examine the associated factors of age at natural menopause with adjusted odds ratios (ORs) and their 95% confidence intervals (CIs) were reported.

**Results:**

The mean age at natural menopause was 48.94 years, with 3.40% of the women experienced premature menopause and 6.75% early menopause. Younger age, higher education, consumption of meat (1–3 days per week) and increased parity were associated with late menopause. Current smoking, underweight, higher physical activity, consumption of sea food (1–3 days per week), fresh eggs (≥4 days per week), fresh fruits (≥1 day per week), taking vitamins, experiencing severe food shortage, earlier age at menarche and older age at first birth were associated with earlier age at natural menopause.

**Conclusions:**

These results suggest that certain factors involved with socio-demographic, lifestyle, dietary and reproductive characteristics are related to the age at natural menopause in Chinese women.

## Introduction

Menopause is an important milestone in women signaling the end of a woman’s reproduction life. The median age at natural menopause is approximately 51 years and varies in different populations [1]. Throughout the last decades interest in the timing of natural menopause has rapidly increased since menopausal age has significant health implications. There is now considerable professional evidence indicating that early or late menopause is linked to a variety of chronic diseases. Early menopause has been reported to be associated with a higher risk of cardiovascular disease [2], all-cause mortality [2], and osteoporosis [3], whilst late menopause has been associated with an increased risk of breast [4], endometrial [5] and ovarian cancer [6]. Thus, identifying the associated factors of menopausal age, especially those that are modifiable, may be important to the prevention of these chronic diseases among postmenopausal women.

Age at natural menopause, as complex trait, is increasingly recognized as driven by a combination of genetic and environmental contributions. Besides, evidence also suggests that over half of the menopausal age variation can be attributed to be non-genetic factors [7]. Among the environmental factors studied previously, smoking has been most consistently proved to be linked to age at natural menopause [8–10]. However, the effect of other potential factors, involving socioeconomic status, dietary intake, lifestyle behaviors, and reproductive history, on the timing of natural menopause is less studied, with no universal consensus [11].

Particularly, the above evidence [8–11] is mainly based on findings from western countries and far fewer studies are conducted in Chinese women. In this paper, with part of cross-sectional data from the China Kadoorie Biobank (CKB) study, we examined the association between age at natural menopause and socio-demographic, lifestyle, dietary and reproductive characteristics in adult women from Zhejiang Province of China.

## Materials and methods

### Study participants

This study was based on data from part of the survey of the CKB study, which was conducted in Tongxiang, a rural county in Zhejiang Province of China between August 2004 and January 2008. Detailed information about the study design, methods and recruitment procedures have been described previously [12–14]. In this study, 57,704 participants, including 33,677 women (58.36%) aged 30–79 years, were recruited. Of 33,677women who participated in this study, 16,601 women were excluded because data on menopause were missing, they were premenopausal or perimenopausal, and they reported surgical menopause. The remaining 17,076 women who had experienced natural menopause, with valid data of menopausal age were included in the study. At the baseline survey, trained health workers collected information on general socio-demographic, lifestyle, dietary and reproductive characteristics of participants using a laptop-based questionnaire, and took physical measurements. All participants provided written informed consent abided by the “Declaration of Helsinki” and the study was approved by the ethics committee of Zhejiang Provincial Center for Disease Control and Prevention.

### Age at natural menopause

Women were asked “Have you had your menopause”. The options were “No”, “Yes, currently”, “Yes, had menopause”. If the answer was ‘Yes, had menopause’, they were then further asked their age of completion of menopause. Women who had hysterectomy, ovariosteresis (unilateral or bilateral) were excluded to avoid surgical menopause. Based on the 10th and 90th percentile, the age at natural menopause was firstly divided into three gruops: ≤44 years, 45–52 years, and ≥53 years. Then, based on relevant literature [15–16], premature menopause (≤40 years) and early menopause (41–44 years) were further defined. Last, to provide more detailed and comparable outcomes, the group of 45–52 years was divided into two groups of 45–49 years and 50–52 years.

### Assessment of exposure variables

Socio-demographic factors included age (≤50, 51–55, 56–60, 61–65, >65 years), marriage status (married or unmarried), highest education (no formal school, primary school, middle school, high school or above), annual household income (<20,000, 20,000–35,000, ≥ 35,000 Yuan). Marriage status was dichotomized as married or unmarried conditions including never married, divorced, separated or widowed.

Lifestyle behaviors included alcohol intake (never, occasional, current regular), smoking status (never, occasional, current regular), tea drinking (never, occasional, current regular), physical activity (low, middle, high), sleep duration (≤6, 7, 8, ≥9 hours), Body mass index (BMI, underweight, normal weight, overweight, obesity). BMI was calculated by weight (kg)/height squared (m^2^). Obesity was defined as BMI ≥28.0 kg/m^2^, overweight 24.0–27.9 kg/m^2^, normal weight 18.5–23.9 kg/m^2^ and underweight <18.5 kg/m^2^. Physical activity calculated as metabolic equivalent task hours (MET-hours/day) spent on work, transportation, housework and non-sedentary recreation, and was analyzed as low, middle and high levels according to the quartiles.

Dietary data over the 12 months preceding the interview were collected. Weekly frequency (<1 day per week, 1–3 days per week, ≥4 days per week) was used to assess the consumption of meat, sea food, fresh eggs, soybean products, fresh fruits, and dairy products. Besides, data about vitamins intake (yes, no), minerals intake (yes, no), and experienced severe food shortage (yes, no) were collected simultaneously.

The reproductive characteristics included age at menarche (≤12, 13–14, 15, 16, ≥17 years), number of live births (≤1, 2, ≥3), age at first birth (<20, 20–24, 25–29, ≥30 years), number of spontaneous abortion (0, 1, ≥2), number of induced abortion (0, 1, ≥2).

### Statistical analysis

The series of characteristics of the participants were described with frequency (N) and percentages (%) according to their age at natural menopause, and were compared using linear-by-linear association chi-square test. By adding all variables from chi-square test, the association between age at natural menopause and characteristics was examined in multinomial logistic regression model, with the menopausal age at 50–52 years as the reference. The reference category of exposure variables can be found in tables. The estimated effect was reported by adjusted odds ratios (ORs) with their 95% confidence intervals (CIs). A p value of <0.05 was considered to be statistically significant. All analyses were performed using SAS statistical package (version 9.2, SAS Institute, Inc., Cary, NC, USA).

## Results

The mean age at natural menopause among the study participants was 48.94 years. 3.40% of the women experienced premature menopause in contrast to approximately doubled (6.75%) women experienced early menopause. The socio-demographic, lifestyle, dietary, reproductive characteristics of participants stratified by age at natural menopause were independently described and shown in Tables 1–4, respectively. We did not observe differences between the two menopausal age groups (≤ 44 years and ≥45 years) regarding alcohol intake, tea drinking, physical activity, consumption of soybean products, dairy products, vitamins, minerals, experienced severe food shortage, number of live birth, age at first birth, number of spontaneous and induced abortion (all P ≥ 0.05).

**Table 1:** Socio-demographic characteristics of women according to age at natural menopause.

Characteristics	Age at natural menopause (years)					P_trend_
	≤40	41–44	45–49	50–52	≥ 53	
**Number of women N (%)**	581 (3.40)	1152 (6.75)	7559 (44.27)	5432 (31.81)	2352(13.77)	
**Age (years) N (%)**						<0.01
**≤50**	95(16.35)	165(14.32)	906(11.99)	101(1.86)	0(0.00)	
**51–55**	79(13.60)	243(21.09)	1925(25.46)	1904(35.05)	482(20.49)	
**56–60**	118(20.31)	276(23.96)	1952(25.82)	1601(29.47)	1033(43.92)	
**61–65**	97(16.69)	209(18.14)	1275(16.87)	907(16.70)	493(20.96)	
**>65**	192(33.05)	259(22.48)	1501(19.86)	919(16.92)	344(14.63)	
**Marriage status N (%)**						0.01
**Married**	471(81.07)	1000(86.81)	6502 (86.02)	4713 (86.76)	2038 (86.65)	
**Unmarried**	110(18.93)	152(13.19)	1057(13.98)	719(13.24)	314(13.35)	
**Highest education N (%)**						0.02
**No formal school**	460(79.17)	875(75.96)	5663(74.92)	3945(72.63)	1621(68.92)	
**Primary school**	100(17.21)	224(19.44)	1611(21.31)	1283(23.62)	602(25.59)	
**Middle school**	16(2.76)	41(3.56)	234(3.10)	169(3.11)	102(4.34)	
**High school or above**	5(0.86)	12(1.04)	51(0.67)	35(0.64)	27(1.15)	
**Annual household income (Yuan) N (%)**						0.04
**<20,000**	177(30.47)	326(28.30)	2000(26.46)	1369(25.20)	610(25.94)	
**20,000–35,000**	219(37.69)	433(37.59)	2978(39.40)	2195(40.41)	935(39.75)	
**≥ 35,000**	185(31.84)	393(34.11)	2581(34.14)	1868(34.39)	807(34.31)	

Differences between the two menopausal age groups (≤ 44 years and ≥45 years) were compared using linear-by-linear association chi-square test.

**Table 2:** Lifestyle characteristics of women according to age at natural menopause.

Characteristics	Age at natural menopause (years)					P_trend_
	≤40	41–44	45–49	50–52	≥ 53	
**Number of women N (%)**	581(3.40)	1152 (6.75)	7559 (44.27)	5432 (31.81)	2352 (13.77)	
**Alcohol intake N (%)**						1.00
**Never**	520(89.50)	1024(88.89)	6755(89.36)	4794(88.25)	2090(88.86)	
**Occasional**	42(7.23)	90(7.81)	557(7.37)	473(8.71)	199(8.46)	
**current regular**	19(3.27)	38(3.30)	247(3.27)	165(3.04)	63(2.68)	
**Smoking status N (%)**						<0.001
**Never**	552(95.01)	1100(95.48)	7281(96.32)	5234(96.35)	2293(97.49)	
**Occasional**	7(1.20)	13(1.13)	111(1.47)	96(1.77)	26(1.11)	
**current regular**	22(3.79)	39(3.39)	167(2.21)	102(1.88)	33(1.40)	
**Tea drinking N (%)**						0.14
**Never**	413(71.08)	819(71.09)	5262(69.61)	3779(69.57)	1600(68.03)	
**Occasional**	103(17.73)	189(16.41)	1357(17.95)	929(17.10)	420(17.86)	
**current regular**	65(11.19)	144(12.50)	940(12.44)	724(13.33)	332(14.11)	
**Physical activity N (%)**						0.05
**Low**	164(28.23)	279(24.22)	2105(27.85)	1480(27.25)	614(26.11)	
**Middle**	275(47.33)	554(48.09)	3608(47.73)	2578(47.46)	1160(49.32)	
**High**	142(24.44)	319(27.69)	1846(24.42)	1374(25.29)	578(24.57)	
**Sleep duration (hours) N (%)**						0.01
**≤6**	123(21.17)	246(21.35)	1446(19.13)	968(17.82)	413(17.56)	
**7**	175(30.12)	315(27.34)	2194(29.02)	1528(28.13)	680(28.91)	
**8**	176(30.29)	396(34.38)	2514(33.26)	1923(35.40)	799(33.97)	
**≥9**	107(18.42)	195(16.93)	1405(18.59)	1013(18.65)	460(19.56)	
**Body mass index N (%)**						<0.001
**Underweight**	64(11.02)	138(11.98)	779(10.31)	411(7.57)	140(5.95)	
**Normal weight**	342(58.86)	638(55.38)	4274(56.54)	3045(56.06)	1327(56.42)	
**Overweight**	144(24.78)	304(26.39)	2018(26.70)	1569(28.88)	696(29.59)	
**Obesity**	31(5.34)	72(6.25)	488(6.45)	407(7.49)	189(8.04)	

Differences between the two menopausal age groups (≤ 44 years and ≥45 years) were compared using linear-by-linear association chi-square test.

**Table 3:** Dietary characteristics of women according to age at natural menopause.

Characteristics	Age at natural menopause (years)					P_trend_
	≤40	41–44	45–49	50–52	≥ 53	
**Number of women N (%)**	581(3.40)	1152 (6.75)	7559 (44.27)	5432 (31.81)	2352 (13.77)	
**Meat N (%)**						<0.001
**<1 day per week**	99(17.04)	172(14.93)	961(12.71)	684(12.59)	261(11.10)	
**1–3 days per week**	275(47.33)	553(48.00)	3721(49.23)	2507(46.15)	1156(49.15)	
**≥4 days per week**	207(35.63)	427(37.07)	2877(38.06)	2241(41.26)	935(39.75)	
**Sea food N (%)**						0.01
**<1 day per week**	371(63.86)	726(63.02)	4655(61.58)	3113(57.31)	1370(58.25)	
**1–3 days per week**	199(34.25)	394(34.20)	2726(36.06)	2187(40.26)	932(39.63)	
**≥4 days per week**	11(1.89)	32(2.78)	178(2.36)	132(2.43)	50(2.12)	
**Fresh eggs N (%)**						0.03
**<1 day per week**	232(39.93)	504(43.75)	3100(41.01)	2079(38.27)	958(40.73)	
**1–3 days per week**	316(54.39)	570(49.48)	3983(52.69)	2882(53.06)	1231(52.34)	
**≥4 days per week**	33(5.68)	78(6.77)	476(6.30)	471(8.67)	163(6.93)	
**Soybean products N (%)**						0.17
**<1 day per week**	99(17.04)	190(16.49)	1127(14.91)	746(13.73)	338(14.37)	
**1–3 days per week**	338(58.18)	696(60.42)	4712(62.34)	3345(61.58)	1469(62.46)	
**≥4 days per week**	144(24.78)	266(23.09)	1720(22.75)	1341(24.69)	545(23.17)	
**Fresh fruits N (%)**						<0.001
**<1 day per week**	359(61.79)	645(55.99)	3768(49.85)	2538(46.72)	1140(48.47)	
**1–3 days per week**	165(28.40)	350(30.38)	2690(35.59)	2041(37.57)	837(35.59)	
**≥4 days per week**	57(9.81)	157(13.63)	1101(14.56)	853(15.71)	375(15.94)	
**Dairy products N (%)**						0.09
**<1 day per week**	575(98.97)	1127(97.83)	7390(97.76)	5304(97.64)	2299(97.75)	
**1–3 days per week**	2(0.34)	17(1.48)	79(1.05)	58(1.07)	24(1.02)	
**≥4 days per week**	4(0.69)	8(0.69)	90(1.19)	70(1.29)	29(1.23)	
**Vitamins intake N (%)**						0.26
**Yes**	24(4.13)	105(9.11)	590(7.81)	457(8.41)	215(9.14)	
**No**	557(95.87)	1047(90.89)	6969(92.19)	4975(91.59)	2137(90.86)	
**Minerals intake N (%)**						0.07
**Yes**	19(3.27)	54(4.69)	393(5.20)	280(5.15)	126(5.36)	
**No**	562(96.73)	1098(95.31)	7166(94.80)	5152(94.85)	2226(94.64)	
**Experienced severe food shortage N (%)**						0.34
**Yes**	401(69.02)	763(66.23)	4954(65.54)	3790(69.77)	1733(73.68)	
**No**	180(30.98)	389(33.77)	2605(34.46)	1642(30.23)	619(26.32)	

Differences between the two menopausal age groups (≤ 44 years and ≥45 years) were compared using linear-by-linear association chi-square test.

**Table 4:** Reproductive characteristics of women according to age at natural menopause.

Characteristics	Age at natural menopause (years)					P_trend_
	≤40	41–44	45–49	50–52	≥ 53	
**Number of women N (%)**	581(3.40)	1152 (6.75)	7559 (44.27)	5432 (31.81)	2352 (13.77)	
**Age at menarche (years) N (%)**						<0.001
**≤12**	28(4.82)	44(3.82)	155(2.05)	129(2.38)	44(1.87)	
**13–14**	83(14.29)	246(21.35)	1238(16.38)	823(15.15)	316(13.43)	
**15**	134(23.06)	257(22.31)	1691(22.37)	1219(22.44)	500(21.26)	
**16**	105(18.07)	230(19.97)	1695(22.42)	1281(23.58)	563(23.94)	
**≥17**	231(39.76)	375(32.55)	2780(36.78)	1980(36.45)	929(39.50)	
**Number of live births N (%)**						0.51
**≤1**	59(10.15)	113(9.81)	635(8.40)	326(6.00)	75(3.19)	
**2**	191(32.87)	462(40.10)	3209(42.45)	2509(46.19)	966(41.07)	
**≥3**	315(54.22)	564(48.96)	3658(48.39)	2560(47.13)	1299(55.23)	
**Age at first birth (years) N (%)**						0.49
**<20**	194(33.39)	415(36.02)	2650(35.06)	2018(37.15)	956(40.65)	
**20–24**	315(54.22)	624(54.17)	4148(54.87)	2884(53.09)	1235(52.51)	
**25–29**	48(8.26)	92(7.99)	628(8.31)	434(7.99)	130(5.53)	
**≥30**	7(1.20)	5(0.43)	63(0.83)	57(1.05)	18(0.77)	
**Number of spontaneous abortion N (%)**						0.08
**0**	534(94.51)	1095(96.14)	7060(94.11)	5112(94.75)	2207(94.32)	
**1**	24(4.25)	35(3.07)	365(4.87)	232(4.30)	115(4.91)	
**≥2**	7(1.24)	9(0.79)	77(1.02)	51(0.95)	18(0.77)	
**Number of induced abortion N (%)**						0.61
**0**	349(61.77)	635(55.75)	4276 (57.00)	3077 (57.03)	1335(57.05)	
**1**	168(29.73)	389(34.15)	2491(33.20)	1794(33.25)	784(33.50)	
**≥2**	48(8.50)	115(10.10)	735(9.80)	524(9.72)	221(9.45)	

Differences between the two menopausal age groups (≤ 44 years and ≥45 years) were compared using linear-by-linear association chi-square test.

After mutual adjustment, the significant associations with premature, early and late menopause in multivariate analysis were presented in Table 5 and the non-significant ones were presented in S1 Table. In this study, women with younger age (56–65 years) were more likely to have late menopause (ORs ranged from 1.38 to 1.65 and the 95% CIs did not include the null); higher education was positively associated with an increased odds of late menopause (ORs ranged from 1.20 to 2.26 and the 95% CIs did not include the null). Current regular smoking was related to increased risk of early menopause by 80% (OR 1.80, 95% CI: 1.22–2.65); compared to normal weight, lower BMI of underweight was associated with a 55% (OR 1.55, 95% CI: 1.25–1.93) higher risk of early menopause; higher physical activity was positively associated with an increased odds of early menopause (ORs ranged from 1.20 to 1.30 and the 95% CIs did not include the null). Consumption of meat (1–3 days per week) was positively associated with late menopause (OR 1.27, 95% CI: 1.08–1.49); consumption of sea food (1–3 days per week), fresh eggs (≥4 days per week), fresh fruits (≥1 day per week), taking vitamins and experienced severe food shortage were inversely associated with earlier age at menopause. Women with earlier age at menarche were more likely to experience premature menopause (OR 2.14, 95% CI: 1.36–3.36) and early menopause (ORs ranged from 1.44 to 1.59 and the 95% CIs did not include the null); two or more live births was positively associated with late menopause (ORs ranged from 1.63 to 1.95 and the 95% CIs did not include the null); older age at first birth (20–24 years) was positively related to premature menopause (OR 1.23, 95% CI: 1.02–1.50).

**Table 5:** Multinomial logistic regression- significant adjusted OR of characteristics and their association with age at natural menopause.

Characteristics	Age at natural menopause (years)			
	≤40 OR(95% CI)	41–44 OR(95% CI)	45–49 OR(95% CI)	≥ 53 OR(95% CI)
**Socio-demographic**				
**Age (years)**				
**≤50**	**4.38(2.88–6.68)**	**5.81(4.12–8.19)**	**5.86(4.59–7.50)**	**0.03(0.004–0.20)**
**51–55**	**0.20(0.14–0.27)**	**0.45(0.36–0.57)**	**0.66(0.58–0.75)**	**0.68(0.56–0.82)**
**56–60**	**0.36(0.28–0.48)**	**0.62(0.50–0.77)**	**0.78(0.70–0.88)**	**1.65(1.40–1.94)**
**61–65**	**0.52(0.40–0.69)**	0.81(0.66–1.01)	**0.87(0.77–0.98)**	**1.38(1.16–1.64)**
**>65**	Ref.	Ref.	Ref.	Ref.
**Highest education**				
**No formal school**	Ref.	Ref.	Ref.	Ref.
**Primary school**	**0.74(0.59–0.94)**	**0.81(0.69–0.96)**	**0.88(0.81–0.96)**	**1.20(1.07–1.35)**
**Middle school**	0.98(0.56–1.70)	1.13(0.79–1.63)	0.96(0.77–1.18)	**1.80(1.38–2.34)**
**High school or above**	1.35(0.47–3.91)	1.59(0.81–3.14)	1.01(0.65–1.57)	**2.26(1.34–3.83)**
**Lifestyle**				
**Smoking status**				
**Never**	Ref.	Ref.	Ref.	Ref.
**Occasional**	0.52(0.22–1.20)	0.62(0.34–1.15)	0.89(0.67–1.17)	**0.52(0.34–0.82)**
**current regular**	1.46(0.89–2.41)	**1.80(1.22–2.65)**	1.21(0.94–1.56)	0.67(0.45–1.00)
**Body mass index**				
**Underweight**	1.20(0.90–1.62)	**1.55(1.25–1.93)**	**1.37(1.20–1.56)**	**0.75(0.61–0.92)**
**Normal weight**	Ref.	Ref.	Ref.	Ref.
**Overweight**	0.85(0.69–1.05)	0.94(0.81–1.09)	0.93(0.85–1.00)	1.04(0.93–1.16)
**Obesity**	0.70(0.47–1.03)	0.84(0.64–1.09)	**0.84(0.73–0.97**)	1.08(0.90–1.30)
**Physical activity**				
**Low**	Ref.	Ref.	Ref.	Ref.
**Middle**	1.10(0.89–1.36)	**1.20(1.02–1.42)**	0.95(0.87–1.04)	1.15(1.00–1.32)
**High**	1.12(0.87–1.45)	**1.30(1.07–1.57)**	**0.89(0.81–0.99)**	1.13(0.97–1.30)
**Dietary**				
**Meat**				
**<1 day per week**	Ref.	Ref.	Ref.	Ref.
**1–3 days per week**	0.93(0.72–1.20)	0.95(0.78–1.16)	1.10(0.98–1.23)	**1.27(1.08–1.49)**
**≥4 days per week**	0.85(0.65–1.13)	0.91(0.74–1.12)	0.99(0.88–1.12)	1.18(1.00–1.40)
**Sea food**				
**<1 day per week**	Ref.	Ref.	Ref.	Ref.
**1–3 days per week**	0.94(0.78–1.14)	**0.83(0.72–0.95)**	**0.84(0.78–0.91)**	1.03(0.93–1.15)
**≥4 days per week**	0.95(0.51–1.79)	1.07(0.71–1.60)	0.92(0.73–1.16)	0.91(0.65–1.27)
**Fresh eggs**				
**<1 day per week**	Ref.	Ref.	Ref.	Ref.
**1–3 days per week**	1.13(0.94–1.36)	0.87(0.76–1.00)	0.96(0.89–1.04)	0.94(0.85–1.05)
**≥4 days per week**	0.78(0.52–1.15)	**0.74(0.56–0.96)**	**0.72(0.62–0.83)**	**0.80(0.66–0.98)**
**Fresh fruits**				
**<1 day per week**	Ref.	Ref.	Ref.	Ref.
**1–3 days per week**	**0.60(0.49–0.73)**	**0.69(0.59–0.79)**	**0.90(0.83–0.97)**	0.94(0.84–1.05)
**≥4 days per week**	**0.54(0.40–0.73)**	**0.73(0.60–0.90)**	0.90(0.81–1.01)	1.05(0.90–1.22)
**Vitamins intake**				
**Yes**	**0.54(0.35–0.84)**	1.19(0.95–1.50)	0.94(0.82–1.07)	1.09(0.92–1.30)
**No**	Ref.	Ref.	Ref.	Ref.
**Experienced severe food shortage**				
**Yes**	**0.80(0.65–0.97)**	**0.82(0.71–0.95)**	**0.83(0.76–0.89)**	1.09(0.97–1.22)
**No**	Ref.	Ref.	Ref.	Ref.
**Reproductive**				
**Age at menarche (years)**				
**≤12**	**2.14(1.36–3.36)**	**1.59(1.09–2.30)**	0.85(0.66–1.08)	0.85(0.60–1.22)
**13–14**	0.95(0.71–1.28)	**1.44(1.18–1.75)**	1.08(0.97–1.22)	0.93(0.79–1.10)
**15**	Ref.	Ref.	Ref.	Ref.
**16**	**0.74(0.56–0.97)**	0.86(0.70–1.04)	0.96(0.87–1.07)	1.07(0.92–1.23)
**≥17**	0.96(0.76–1.21)	0.86(0.71–1.02)	1.01(0.92–1.11)	1.12(0.98–1.28)
**Number of live births**				
**≤1**	Ref.	Ref.	Ref.	Ref.
**2**	**0.49(0.34–0.68)**	**0.64(0.49–0.84)**	**0.84(0.71–0.98)**	**1.95(1.48–2.58)**
**≥3**	**0.38(0.28–0.53)**	**0.54(0.43–0.69)**	**0.69(0.60–0.80)**	**1.63(1.25–2.13)**
**Age at first birth (years)**				
**<20**	Ref.	Ref.	Ref.	Ref.
**20–24**	**1.23(1.02–1.50)**	1.07(0.93–1.23)	**1.10(1.02–1.19)**	0.96(0.87–1.07)
**25–29**	1.33(0.93–1.91)	1.01(0.77–1.33)	1.11(0.96–1.28)	**0.75(0.60–0.94)**
**≥30**	1.39(0.60–3.21)	0.42(0.16–1.07)	0.89(0.61–1.29)	0.83(0.48–1.45)

Bold values represent significant results.

Ref.: reference group, OR: odds ratio, CI: confidence interval.

## Discussion

To our knowledge, this is the one of very few studies to comprehensively examine the relationship of various factors and the timing of natural menopause in Chinese women. The overall mean age at natural menopause was 48.94 years in this population, which is earlier than other Chinese studies results (49–50 years) [17–18], and even earlier than the values previously observed in developed countries (≥51 years) [1, 19–22]. The variation in menopausal age may be due to the differences in methodological issue, race, lifestyle behavior, and additionally, in this study we proposed that the lower socioeconomic status of our participants, which were recruited from rural area, might be responsible for the earlier age at natural menopause. Furthermore, our findings showed that over 10% of the women had premature or early menopause, which is slightly higher than the results shown in a multinational study of 51,450 postmenopausal women [23]. Considering the large population of Chinese menopausal women [24], the issue of earlier onset of natural menopause needs more attention.

Consistent with many studies [10, 25–27], our findings showed that the socioeconomic characteristics of higher education was associated with a later age at natural menopause. According to previous reports [17–18], the education level may affect the onset of menopause through physiological effects on lifestyle behaviors and reproductive health. Compared to education level, little is known about the relationship of age and the timing of menopause. In our study, younger age (56–65 years) was related to later menopause, which confirms the findings from another CKB study of over 300,000 women [26]. However, a Japanese study revealed that older generation was associated with earlier onset of natural menopause [19].

In the present study, current regular smoking was associated with increased risk of early menopause. The direction of our results regarding smoking is in agreement with prior studies [8, 10, 18–19, 28]. Recently, several studies have observed a dose-response relationship between smoking amount and the earlier onset of menopause [9, 29]. Although the mechanism by which smoking affects earlier menopause is unclear, based on current evidence, the toxic components of cigarette smoke may destroy primordial oocytes, decrease oestrogen production and increase the 2-hydroxylation [30–31]. Our results indicated that women with underweight were more likely to have early menopause compared with women with normal BMI, which is in line with previous studies [9, 32–33]. In terms of biological mechanism, Ogden et al. proposed that in the postmenopausal period, women with a high body mass index had higher circulating levels of estrogen than leaner women [34]. However, in general, the association between BMI and age at natural menopause has been controversial [18, 35–36] and needs further investigations in well-designed studies. As for the physical activity and age at natural menopause, we found that higher physical activity was positively associated with an increased odd of early menopause, which is consistent with some previous studies [10, 25]. Based on current evidence, physical activity would result in significant decreases in serum estrogens, which could lead to early menopause [37]. However, the finding has not been wholly consistent across studies, since some authors found a delaying effect [17], whereas one study observed no effect on menopause [28].

In the present study, various dietary factors were associated with age at natural menopause. Our data showed that meat consumption (1–3 days per week) was positively associated with late menopause, which is consistent with former results [28, 38]. Fruit consumption (≥1 days per week) was inversely linked to earlier age at menopause, which seems to confirm the findings from Shanghai Women’s Health Study (SWHS) [17]. An inverse association of sea food (1–3 days per week) and fresh eggs (≥4 days per week) consumption with early menopause was also observed in our data. To some extent, this is in line with the effects of high protein intake on delayed onset of natural menopause reported in prior studies [17, 28], although other nutrients may make a difference. As for the nutritional supplements intake, there has been little research on the relation to menopausal age. Our results suggested that use of vitamins was associated with decreased risk of premature menopause, while a recent European study did not find a significant association in fully-adjusted model [27]. Besides, our data indicated that women who experienced severe food shortage were less likely to have an earlier age at natural menopause than women who did not, which has not been specifically studied before.

With regard to the reproductive factors, we observed an association between earlier age at menarche and increased odds of earlier age at natural menopause, consistent with most previous studies [18, 23]. SWHS reported that later age at menarche resulted in later menopause [17], but no such association was evident in our results. Consistent with previous studies [9, 11, 17], between increased parity and late menopause we found a positive association. An observation that is also consistent with the theory that natural menopause occurs after oocytes have been sufficiently depleted [39]. Meanwhile, many studies also reported that nulliparous women were at greater risk of early natural menopause [18–19, 23]. Our data suggested that women who reported an older age at first birth tended to have premature menopause, which is in agreement with former data [17], but not with others [28, 40].

The strengths of the present study included the large sample size and comprehensive evaluation of various factors on the age at natural menopause among Chinese women. However, there are several potential limitations in this study. First, the data of outcome and exposure variables were collected mostly based on self-reported and so may be subject to misclassification and recall bias. However, previous studies have indicated that menopausal age by recall is reasonably well [41–43]. Second, as the participants were selected from a rural area in Zhejiang Province, caution is needed in generalizing our findings to the whole Chinese women population. Third, with the cross-sectional data, we could not provide causal association between the various factors and age at natural menopause.

## Conclusions

In conclusion, our results suggested that certain factors involved with socio-demographic, lifestyle, dietary and reproductive characteristics are related to the age at natural menopause in Chinese women. Further longitudinal studies are needed to clarify these associations.

## Supporting information

S1 Table Multinomial logistic regression- non-significant adjusted OR of characteristics and their association with age at natural menopause. (DOCX)

S1 File The data set used in the study. (SAV)
