# Supplementary material for: Age at natural menopause and associated factors in adult women: Findings from the China Kadoorie Biobank study in Zhejiang rural area
Source: PLoS One. 2018 Apr 18;13(4):e0195658. doi: 10.1371/journal.pone.0195658 (PMC5905992; doi:10.1371/journal.pone.0195658)
Supplement: S1 Table — (DOCX) [file pone.0195658.s001.docx]

| Supplementary table 1 Multinomial logistic regression- non-significant adjusted OR of characteristics and their association with age at natural menopause | | | | |
| --- | --- | --- | --- | --- |
| Characteristics | Age at natural menopause (years) | | | |
|  | ≤40  OR(95% CI) | 41-44  OR(95% CI) | 45-49  OR(95% CI) | ≥ 53  OR(95% CI) |
| Socio-demographic |  |  |  |  |
| Marriage status |  |  |  |  |
| Married | 0.79(0.62-1.00) | 1.07(0.87-1.30) | 0.91(0.81-1.01) | 1.13(0.97-1.31) |
| Unmarried | Ref. | Ref. | Ref. | Ref. |
| Annual household income (Yuan) |  |  |  |  |
| <20,000 | Ref. | Ref. | Ref. | Ref. |
| 20,000-35,000 | 0.81(0.66-1.01) | 0.85(0.72-1.00) | 0.92(0.84-1.01) | 1.02(0.90-1.15) |
| ≥ 35,000 | 0.88(0.70-1.11) | 0.95(0.80-1.12) | 0.97(0.88-1.06) | 1.04(0.91-1.18) |
| Lifestyle |  |  |  |  |
| Alcohol intake |  |  |  |  |
| Never | Ref. | Ref. | Ref. | Ref. |
| Occasional | 0.92(0.66-1.28) | 0.93(0.73-1.19) | **0.84(0.74-0.96)** | 0.94(0.79-1.13) |
| current regular | 1.09(0.67-1.78) | 1.04(0.72-1.51) | 1.07(0.87-1.31) | 0.87(0.65-1.18) |
| Tea drinking |  |  |  |  |
| Never | Ref. | Ref. | Ref. | Ref. |
| Occasional | 1.09(0.86-1.38) | 0.94(0.79-1.13) | 1.04(0.95-1.15) | 1.09(0.96-1.24) |
| current regular | 0.83(0.62-1.10) | 0.91(0.75-1.11) | 0.94(0.84-1.04) | 1.12(0.97-1.29) |
| Sleep duration (hours) |  |  |  |  |
| ≤6 | Ref. | Ref. | Ref. | Ref. |
| 7 | 0.95(0.74-1.22) | 0.83(0.68-1.00) | 0.96(0.86-1.07) | 1.07(0.93-1.25) |
| 8 | 0.84(0.65-1.08) | 0.88(0.73-1.06) | **0.88(0.79-0.98)** | 1.02(0.88-1.18) |
| ≥9 | 0.96(0.72-1.28) | 0.82(0.66-1.02) | 0.94(0.83-1.05) | 1.12(0.95-1.32) |
| Dietary |  |  |  |  |
| Soybean products |  |  |  |  |
| <1 day per week | Ref. | Ref. | Ref. | Ref. |
| 1-3 days per week | 0.84(0.66-1.08) | 0.90(0.75-1.08) | 0.98(0.89-1.09) | 0.98(0.84-1.13) |
| ≥4 days per week | 0.92(0.70-1.23) | 0.87(0.70-1.08) | 0.93(0.82-1.05) | 0.92(0.78-1.09) |
| Dairy products |  |  |  |  |
| <1 day per week | Ref. | Ref. | Ref. | Ref. |
| 1-3 days per week | 0.41(0.10-1.69) | 1.62(0.93-2.82) | 1.07(0.76-1.52) | 0.95(0.58-1.55) |
| ≥4 days per week | 0.70(0.25-1.94) | 0.58(0.28-1.23) | 0.99(0.72-1.37) | 0.90(0.57-1.43) |
| Minerals intake |  |  |  |  |
| Yes | 0.75(0.47-1.22) | 0.92(0.68-1.25) | 1.05(0.90-1.24) | 1.04(0.83-1.29) |
| No | Ref. | Ref. | Ref. | Ref. |
| Reproductive |  |  |  |  |
| Number of spontaneous abortion |  |  |  |  |
| 0 | Ref. | Ref. | Ref. | Ref. |
| 1 | 1.02(0.66-1.57) | 0.71(0.50-1.03) | 1.16(0.98-1.37) | 1.14(0.91-1.44) |
| ≥2 | 1.15(0.52-2.56) | 0.73(0.35-1.55) | 1.11(0.77-1.58) | 0.83(0.48-1.43) |
| Number of induced abortion |  |  |  |  |
| 0 | Ref. | Ref. | Ref. | Ref. |
| 1 | 0.86(0.71-1.04) | 1.05(0.92-1.21) | 1.00(0.93-1.08) | 1.04(0.93-1.16) |
| ≥2 | 0.83(0.60-1.15) | 1.05(0.84-1.31) | 1.01(0.90-1.15) | 1.04(0.87-1.23) |
| Bold values represent significant results.  Ref.: reference group, OR: odds ratio, CI: confidence interval. | | | | |
